# New Insights into the Latest Advancement in α-Amylase Inhibitors of Plant Origin with Anti-Diabetic Effects

**DOI:** 10.3390/plants12162944

**Published:** 2023-08-14

**Authors:** Hamdy Kashtoh, Kwang-Hyun Baek

**Affiliations:** Department of Biotechnology, Yeungnam University, Gyeongsan 38541, Republic of Korea; hamdy_kashtoh@ynu.ac.kr

**Keywords:** alpha-amylase, anti-diabetic activity, postprandial hyperglycemia, extract, natural compounds, type 2 diabetes

## Abstract

The rising predominance of type 2 diabetes, combined with the poor medical effects seen with commercially available anti-diabetic medications, has motivated the development of innovative treatment approaches for regulating postprandial glucose levels. Natural carbohydrate digestion enzyme inhibitors might be a viable option for blocking dietary carbohydrate absorption with fewer side effects than manufactured medicines. Alpha-amylase is a metalloenzyme that facilitates digestion by breaking down polysaccharides into smaller molecules such as maltose and maltotriose. It also contributes to elevated blood glucose levels and postprandial hyperglycemia. As a result, scientists are being urged to target α-amylase and create inhibitors that can slow down the release of glucose from carbohydrate chains and prolong its absorption, thereby resulting in lower postprandial plasma glucose levels. Natural α-amylase inhibitors derived from plants have gained popularity as safe and cost-effective alternatives. The bioactive components responsible for the inhibitory actions of various plant extracts have been identified through phytochemical research, paving the way for further development and application. The majority of the findings, however, are based on in vitro investigations. Only a few animal experiments and very few human investigations have confirmed these findings. Despite some promising results, additional investigation is needed to develop feasible anti-diabetic drugs based on plant-derived pancreatic α-amylase inhibitors. This review summarizes the most recent findings from research on plant-derived pancreatic α-amylase inhibitors, including plant extracts and plant-derived bioactive compounds. Furthermore, it offers insights into the structural aspects of the crucial therapeutic target, α-amylases, in addition to their interactions with inhibitors.

## 1. Introduction

**Figure 1:**
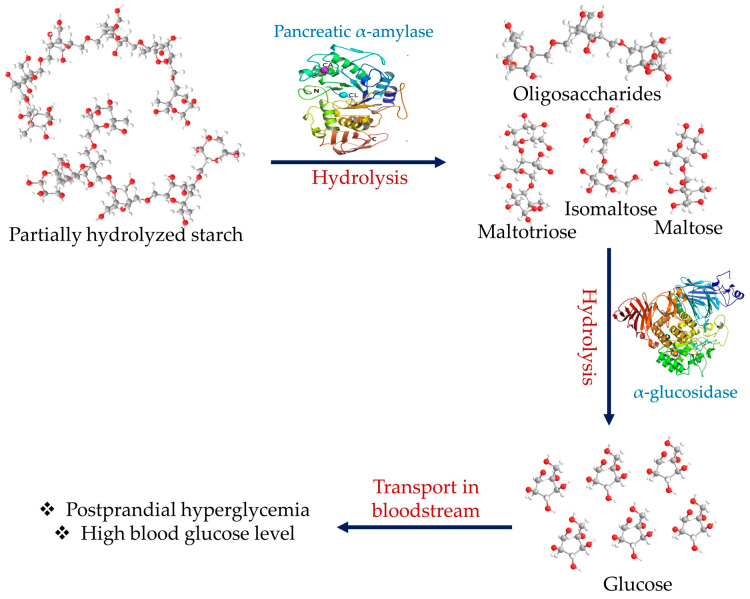
Schematic depiction of the involvement of α-amylase in postprandial hyperglycemia linked with starch hydrolysis.

Diabetes mellitus is a group of metabolic disorders marked by persistent increases in blood glucose levels. This condition is caused by a defect in insulin production, insulin function, or both factors. Insulin insufficiency and its resistance to the intended target tissues cause abnormalities in the metabolic processing of carbohydrates, lipids, and proteins [1,2,3]. Diabetes is linked to several short- and long-term health ramifications. Acute health problems include diabetic ketoacidosis, malignant hyperthermia-like syndrome with rhabdomyolysis, and hyperosmolar hyperglycemia, all of which carry a significant risk of morbidity and fatality in the near term [4,5]. The long-term ramifications might entail hypertension, lipid disorders, retinopathy, renal malfunction, nonalcoholic fatty liver disease, neurological diseases, cardiovascular and atherosclerotic problems, and others [4,5]. The most prevalent signs of hyperglycemia include polyuria, excessive thirst, unexplained weight loss, hyperphagia, and a lack of visual acuity [6]. Depending on the mode of its manifestation, there are several different types of diabetes, which include: (1) type 1 diabetes (T1D), which is caused by the destruction of pancreatic β-cells by the body’s immune system; (2) type 2 diabetes (T2D), which is characterized primarily by reduced insulin receptor sensitivity; (3) gestational diabetes (GD), which is recognized in the second or third trimester of pregnancy but was not concisely identified as diabetes prior to gestation; and (4) particular kinds of diabetes caused by different factors [7]. Diabetes mellitus is a major global disease, affecting over half a billion individuals in 2021, and the number of adults affected is expected to reach nearly 800 million by 2045 [8,9]. T2D, which is the most prevalent type (~90%), is a metabolic disorder that involves carbohydrate, lipid, and protein metabolic abnormalities as well as deficiencies in insulin secretion, typically accompanied by insulin resistance [10]. These chronic metabolic disorders may result in neuropathy, retinopathy, angiopathy, and nephropathy [11]. In this perspective, carbohydrate-digesting enzyme inhibition is regarded as a therapeutic strategy for the management and treatment of T2D. The most abundant carbohydrate in meals is starch, which is composed of two distinguished polysaccharides: one is linear with α-(1→4) glycosidic bonds (amylose), and the other is branched with α-(1→6) glycosidic bonds (amylopectin) [12]. Pancreatic α-amylase (EC 3.2.1.1) is the most essential digestive enzyme. It is a calcium-based metalloenzyme that acts as a catalyst and facilitates the hydrolysis of the α-1,4 glycosidic bonds of polysaccharide molecules such as amylose, amylopectin, glycogen, and other maltodextrins and is accountable for the majority of starch digestion in humans. Another digestive enzyme, α-glucosidase or maltase (EC 3.2.1.20), catalyzes the final phase of carbohydrate digestion, acting on 1,4-alpha bonds to generate glucose (Figure 1) [13].

Scientific investigations have shown the association between the activities of human pancreatic α-amylase (HPA) and postprandial glucose levels, highlighting the importance of lowering postprandial hyperglycemia (PPHG) in the management of T2D [14]. The capacity of α-amylase enzyme inhibitors to prevent the digestion and absorption of dietary starch has led to their being categorized as starch blockers. Nevertheless, α-pancreatic amylase activity inhibition should be moderated to avoid bacterial fermentation of non-digested carbohydrates in the colon caused by excessive suppression of the activity of this enzyme, which gives rise to diarrhea and flatulence [15]. There are already certain diabetes medications that work primarily by limiting carbohydrate digestion and absorption. The first α-glucosidase inhibitor accessible for diabetic therapy was acarbose, a microbial inhibitor that inhibits α-amylase, maltase, and sucrase (EC 3.2.1.48) activities. Voglibose is a novel bacterial α-glucosidase inhibitor that suppresses maltase, isomaltase (EC 3.2.1.10), and sucrase activities, whereas miglitol is a 1-deoxynojirimycin derivative that functions by inhibiting the activities of glucoamylase (EC 3.2.1.3), sucrase, and isomaltase [16,17]. Although these medicines are effective in stabilizing postprandial blood glucose levels in a large number of individuals, they are frequently linked with substantial gastrointestinal side effects [16]. Furthermore, the undesirable clinical outcomes associated with marketed anti-hyperglycemic drugs are partly to blame for the common medication failure to comply that occurs in diabetes patients [18]. Because of the significant side effects of such medications, researchers have been looking for substitute therapies with minimal or no harmful effects. Although these anti-diabetic medicines are commercially available, several attempts have been made to produce non-cytotoxic anti-diabetic synthetic molecules [19,20,21]. From such a perspective, herbal chemicals intend to offer milder techniques of controlling metabolic problems and have been employed in traditional medical systems such as Indian Ayurveda (alternative medicine), Chinese herbal medicines, and Arabic Unani (traditional medicine used by the Muslims) since ancient times [22]. As a result, there is evidence suggesting the potentially helpful impact of a vast variety of medicinal herbs in T2D management [23]. Aside from their efficiency, herbal therapies appear to have few side effects and offer a cost-effective alternative to ingested commercial hypoglycemic medications. The World Health Organization (WHO) suggested in 1990 that extensive studies be conducted on the positive benefits of these plants [24]. In this regard, the current review highlights research on the α-amylase inhibitory action of plants and their phytochemical components, which may be effective in the treatment of diabetes. The review was conducted utilizing a scientific database composed of web search engines, including SciFinder, Scopus, Google Scholar, PubMed, and Science Direct, to examine published material between 2019 and 2023. Search criteria included ‘amylase inhibitor’, ‘anti-diabetic characteristics’, ‘plant extracts’, and ‘pancreatic amylase’. The latest articles related to pancreatic amylase (porcine and human) inhibition by plant extracts were also included. Medical plants having a folkloric background and α-amylase activity met the inclusion requirements.

## 2. Alpha-Amylase Structure and Mechanism of Action

In the human diet, starch serves as the main energy source. Dietary sugars and starch are broken down to glucose by α-glucosidase and α-amylase enzymes. α-amylase metalloenzymes can be found in the saliva and pancreatic juice and are members of the glycoside hydrolase family 13 (GH13) [25,26,27,28]. Metalloenzymes are a diverse collection of enzymes that utilize a metal cation as a co-factor in the enzyme’s active site. These enzymes stimulate a wide range of reactions, including hydrolytic activities [29]. α-amylase is a metalloenzyme that needs an important calcium ion for structural integrity and is activated by chloride ions [30,31,32]. Although this family’s overall amino acid sequence homology is low, it has short regions of highly conserved residues, and structural investigations have shown that its members do have comparable three-dimensional structures [33]. These isozymes are members of a multigene family located on chromosome 1 that is controlled such that the various isozymes are only expressed in the pancreas or salivary glands [34]. The genes AMY1 and AMY2 produce both salivary and pancreatic α-amylase isozymes, each of which has 496 amino acids in one polypeptide chain [35,36,37]. Before starch is absorbed, salivary and pancreatic α-amylases hydrolyze it in the mouth and small intestine [25,38,39]. Through the cleavage of 1-4-α-glycosidic bonds, these isozymes hydrolyze carbohydrate polymers into shorter oligomers, such as maltose, a-limit dextrins, and maltotriose. An early partial cleavage into smaller oligomers (10–30%) is provided by the salivary isozyme [37,40]. When partially digested saccharides enter the gut, pancreatic amylase, which is produced in the pancreas and secreted into the lumen, extensively hydrolyzes them into smaller oligosaccharides [38]. Subsequently, α-glucosidases located in the brush border hydrolyze the α-amylase products further into glucose in the lumen of the small intestine [25,39,41,42]. Afterward, glucose transporters take the glucose from the intestinal mucosa and transport it into the blood circulation [42].

**Figure 2:**
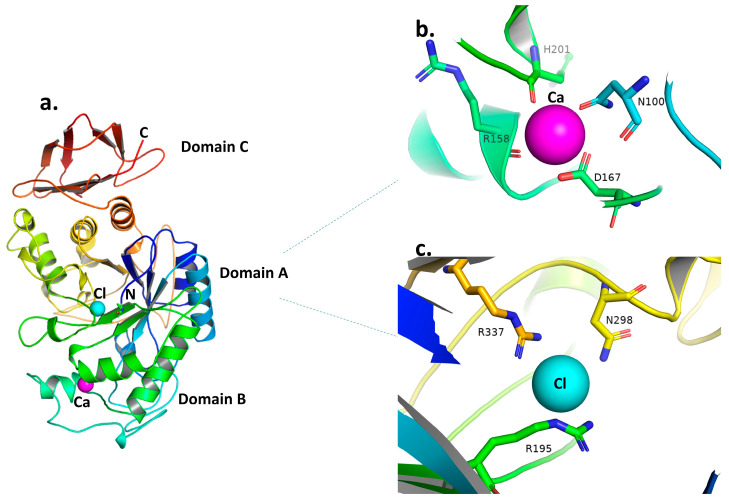
(**a**) Ribbon diagram of the structure of human pancreatic α-amylase as a representative for GH13 α-amylase. The three structural domains (A, B, and C) are indicated and colored as follows: Domain A in blues, greens, yellows, and oranges; Domain B in lime green and pale cyan; and Domain C in red. The N and C terminals are colored blue and red, respectively. The calcium and chloride ions are shown as magenta and cyan spheres, respectively. (**b**) Human pancreatic α-amylase calcium binding site; the sticks represent residues making ligand interactions with calcium, which is represented as a magenta sphere. (**c**) Human pancreatic α-amylase chloride binding site; the sticks represent residues making ligand interactions with chloride, which is represented as a cyan sphere. From the structure, (**a**–**c**) were adopted with PDB entry code 1HNY [33] and produced using PyMol [47].

**Figure 3:**
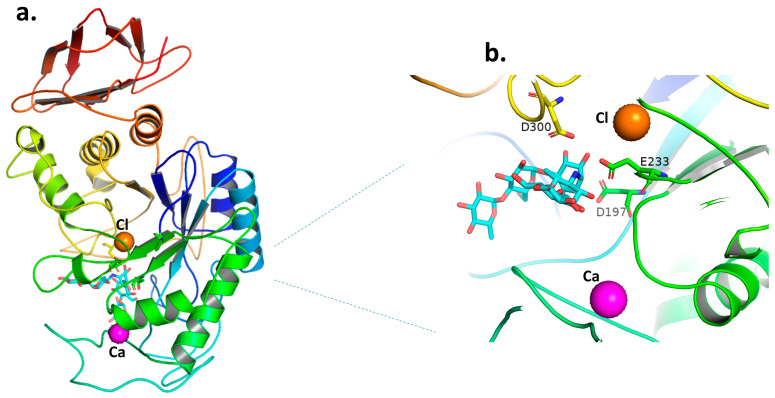
(**a**) The structure of the human pancreatic α-amylase/acarbose complex is depicted as a ribbon diagram. The chloride and calcium ions are represented as orange and magenta spheres, respectively, in (**a**,**b**). (**b**) The active site of the human pancreatic α-amylase/acarbose complex; residues located within a 4-A° radius of acarbose are represented as sticks (the catalytic triad of D197-D300-E233). The acarbose, shown as sticks in (**a**,**b**), is colored cyan. From the structure, (**a**,**b**) were adopted with PDB entry code 1B2Y [48] and produced using PyMol [47].

The 3D structures of the α-amylase enzymes from human saliva, pancreas, and pig pancreas have been determined using X-ray crystallography [33,43,44]. These enzymes’ architectures are all quite similar to one another. Three structural domains (A, B, and C) make up mammalian amylases, the biggest of which, Domain A, creates a standard core of (β-α)_8_ barrel fold (Figure 2a), one end of which is positioned at the crucial active site residues (the catalytic triad of two aspartate (D197 and D300) and one glutamate (E233) residue) (Figure 3b). The active site is located in a substantial cleft that separates the carboxyl termini of the A and B domains. A bound chloride ion is present in Domain A as well, and it has been long recognized to activate amylase [32,45]. The bound chloride ion was found to form ligand interactions with R195, N298, and R337 in close proximity to the active site (Figure 2c) [33]. Domain B, the smallest domain, creates a calcium-binding site against Domain A’s β-barrels. The bound Ca^2+^ ion in Domain B, which also borders the area of the active site, is likely crucial in preserving the active site region protein configuration. N100, R158, D167, and H201, which make ligand interactions with calcium, may greatly contribute to its function (Figure 2b). Anti-parallel β-structure makes up Domain C, which is only tangentially related to Domains A and B. Notably, a stable pyrrolidone derivative is created by post-translational alteration of human pancreatic a-amylase’s N-terminal glutamate residue, which may offer defense against other digesting enzymes. It does not seem plausible that the molecule’s N-terminal Domain C will directly contribute to the catalytic process as it has a weaker connection to Domains A and B [33,46].

The catalytic processes in amylases are excellent representatives of a twofold displacement mechanism, in which the carboxyl groups of Asp and Glu residues serve as acid-base catalysts and a nucleophilic reactant in the creation of a covalent intermediate during the catalysis sequence. Through the use of a charge relay mechanism, a Cl^−^ ion may facilitate the protonation of a catalytically significant carboxyl group, leading to the activation [49,50,51]. With maltotriose and maltose as the main short oligomer yields (very little production of glucose), *α*-amylase is an endoenzyme that attacks linear sections of amylose and amylopectin several times [52,53,54]. According to kinetic studies, the pig pancreatic enzyme’s active site may take up to five glucose molecules, meaning that there are five sub-sites where glucose molecules can bind [52]. Several three-dimensional structures demonstrated the presence of multiple sites, but the pig pancreatic amylase structure complexed with acarbose showed a sixth site [44]. The target of anti-diabetic drugs such as *α*-amylase and *α*-glucosidase inhibitors is to reduce postprandial hyperglycemia, with the most widely used ones being acarbose, voglibose, and miglitol. However, when used in therapy, the inhibitors of *α*-glucosidase and *α*-amylase have been associated with gastrointestinal adverse effects such as diarrhea, bloating, and flatulence. The quest for the development of novel *α*-amylase and *α*-glucosidase inhibitors is therefore crucial for the management of PPHG in T2D. Such insights into *α*-amylase’s structure can be used to better understand the interaction of *α*-amylase with various inhibitors in order to develop anti-diabetic medicines with fewer adverse effects.

## 3. Plant Extracts as an α-Amylase Inhibitor Source

Several traditional medicines, in addition to the existing therapeutic options, have been promoted for diabetes therapy. Traditional plant medicines are used to manage a broad variety of diabetic symptoms all around the world. It is well acknowledged that plant remedies have fewer negative effects than modern pharmaceuticals [55,56,57,58,59,60], as well as being less expensive, driving both the public and healthcare institutions to investigate natural medicinal goods as alternatives to synthetic medications. As a consequence, studies on traditional medicinal herb-derived substances have grown in importance [61,62]. Several folkloric/medicinal plant extracts have been shown to possess potent α-amylase inhibition activity; however, further animal studies are needed to confirm their hypoglycemic physiological impact. Many studies investigated the potential role of medicinal plants in inhibiting α-amylase enzymes (Table 1). Among the latest plant extracts investigated in the literature that are featured in this study, *Prosopis cineraria* (L.), *Terfezia claveryi*, *Chenopodium album* L., and *Salvia lavandulifolia Vahl* have the highest potential to inhibit α-amylase enzymes (Table 1).

A recent study investigated the hypoglycemic effect of edible plant leaves from Palestine that are used as folkloric anti-diabetic remedies [67]. The lipophilic and hydrophobic fractions of these plants were tested against the porcine pancreatic α-amylase enzyme. The hydrophilic fractions of *Centaurea iberica* and *Cichorium endivia* showed the highest α-amylase activity with IC_50_ values of 12.33 μg/mL and 9.96 μg/mL, respectively. Furthermore, the highest α-amylase inhibition effect for lipophilic fractions was observed for *Sisymbrium irio* and *Arum palaestinum,* with IC_50_ values of 7.72 μg/mL and 25.3 μg/mL, respectively. Additionally, rice extract’s biological activity against α-amylase was investigated in Thailand, and the study showed that brown rice extract as well as the rice’s volatile compounds, identified as vanillin and vanillyl alcohol, have high inhibitory effects against α-amylase [68]. It is notable to mention that a synergy effect was noticed on α-amylase inhibition activity when a combination of black, red, and white rice extracts along with vanillin and vanillyl alcohol was used.

*Rhus coriaria* L. leaves and fruits are an important folk medicine that is used in Turkey for the treatment of diabetes [104]. Gök et al. studied the effect of *R. coriaria* ethanol extracts of leaf and fruit on α-amylase inhibitory activity in an attempt to isolate the active compounds against α-amylase [70]. The ethyl sub-extract of *R. coriaria* showed good α-amylase inhibition activity with an IC_50_ of 20.81 μg/mL against 26.99 μg/mL for acarbose. The study showed the successful isolation of several compounds; among them, penta-*O*-galloyl-β-glucopyranose, one of the main compounds in leaf and fruit extracts, had α-amylase inhibition activity with an IC_50_ of 6.32 μM compared to 10.69 μM for acarbose. Peanuts (*Arachis hypogaea*), another edible food, were investigated for their α-amylase inhibition property [72]. Various organic extracts of peanuts were investigated, and it was found that peanut seed ethanol extract has an α-amylase suppressing ability with an IC_50_ of 0.61 μg/mL, close to 0.31 μg/mL for acarbose, and the least cytotoxicity with an LC_50_ of 413.9 μg/mL. *Melilotus officinalis* (yellow sweet clover) is a medicinal plant typically employed in Asia and Europe and has been used as an anti-inflammatory traditional medicine [105,106]. As *M. officinalis* contains a high amount of coumarin derivatives, its extracts were clinically tested for the treatment of diabetic foot [107]. Paun et al. studied the polyphenolic-rich extracts of *M. officinalis* and their anti-diabetic activity [76]. *M. officinalis* polyphenolic-rich extracts displayed notable α-amylase inhibitory activity with an IC_50_ of 1.30 μg/mL, while acarbose showed an IC_50_ of 17.68 µg/mL, suggesting that it could be a good candidate for developing a natural anti-diabetic food supplement.

*Solanum* species are a rich source of traditional medicine remedies as antipyretic, anti-inflammatory, antioxidant, and anti-diabetic agents [108,109,110]. Ju’a-açu fruit (*Solanum oocarpum*), also known as Brazilian sunberry, has an alkaloid composition and has been reported to have anti-diabetic activity [111]. Saraswathi et al. studied the phytoconstituents of the ethanolic and aqueous extracts of *Solanum virginianum* dried fruits for their anti-diabetic activity [92]. The study showed that both the ethanolic and aqueous fruit extracts have α-amylase inhibition activity, with the aqueous extract having higher enzymatic inhibition activity (54.12–86.80%) than the ethanolic extract (23.07–81.61%). 

In China, raspberry leaves are consumed as tea, and it has been reported to have anti-diabetic activity [112]. In a recent study, Li et al. investigated the inhibitory activity of raspberry leaf tea (*Rubus corchorifolius* L.) (RLT) extract digestive enzymes and found potent α-amylase inhibition activity for its ethanolic, methanolic, and aqueous extracts with an IC_50_ of 1.26 mg/mL, 1.47 mg/mL, and 4.39 mg/mL, respectively, against an IC_50_ of 5.12 mg/mL for acarbose aqueous extract [102]. The study identified the major inhibitors responsible for the extract activity as epigallocatechin gallate (EGCG), isovitexin, rutin, isoorientin, procyanidin, delphinidin-3-*O*-glucoside, dihydromyricetin, and procyanidin C3. The authors conducted additional molecular docking analyses and discovered these inhibitors interact with the digestive enzyme through hydrogen bonds or van der Waals forces, resulting in the retardation of enzyme activity [102]. Mikailu et al. studied the effect of the stem bark *Maesobotrya dusenii* Hutch methanol extract on α-amylase inhibition activity. The authors found that all the extracts showed significant amylase activity with 56.7% inhibition in a dose-dependent manner. *Maesobotrya dusenii* Hutch crude methanol extract showed α-amylase activity with an IC_50_ of 24 μg/mL against 28 μg/mL for acarbose. 

*Sterculia nobilis* Smith seeds, leaves, and nuts are used to prepare several food dishes in China. It is commonly native to Vietnam, Indonesia, Japan, and south China and is usually utilized to heal gastrointestinal and circulatory problems [113,114,115]. The dark red shell of *S. nobilis* Smith fruit (pericarp) has been investigated for its inhibition activity against digestive enzymes [103]. The study showed that the pericarp ethyl acetate fraction has a potent uncompetitive α-amylase activity, with an IC_50_ of 13.55 μg/mL against 19.45 μg/mL for acarbose. Spectroscopic methods were used to elaborate the mechanism of α-amylase inhibition, and the results showed the ethyl acetate fraction alters the enzyme’s secondary structure and tryptophan/tyrosine residue microenvironment, resulting in enzyme activity inhibition [103]. The early investigation of these traditional medicinal plant extracts demonstrated promising α-amylase inhibitory activity, but more research is needed to confirm their anti-diabetic impact.

*Prosopis cineraria* (L.) Druce pods are usually used in diets as a vegetable in the Indian subcontinent and have been reported to have anti-diabetic properties [116]. Kumar et al. studied the anti-diabetic effect of *P. cineraria* pod extracts in vitro as well as in vivo (Table 2) [78]. The study shows that n-butanol fractions from the pods have potent α-amylase inhibition activity with an IC_50_ of 22.01 μg/mL against 39.26 μg/mL for acarbose. The n-butanol fraction was investigated for toxicity and found to be non-toxic when the mice ingested an oral dose of the fraction up to 2000 mg/kg. Further studies are required to investigate the *P. cineraria* pods as a promising anti-diabetic candidate. A recent study showed *Terfeziaclaveryi* (truffle, a fungus that grows wildly in the desert) methanol extract to have an α-amylase inhibition activity of 38.7 μg/mL, which is higher when compared to 45.3 μg/mL of acarbose [80]. The in vivo anti-diabetic activity showed that a 200 mg/kg dose of *Terfeziaclaveryi* methanol extract reduced the fasting plasma glucose level. *Chenopodium album* L. is another anti-diabetic herbal remedy candidate that showed promising α-amylase activity from its methanolic and aqueous root extracts [117,118]. *C. album* aerial parts’ alkaloid fraction (CAAF) showed a more potent α-amylase enzyme inhibition activity than acarbose, with an IC_50_ of 122.18 μg/mL and 812.83 μg/mL, respectively [91]. The CAAF fraction did not produce severe toxicity in vivo and showed promising anti-diabetic activity in a dose-dependent manner. The study suggests that the CAAF acts primarily as an α-amylase inhibitor. *Salvia lavandulifolia* Vahl is commonly found in the Mediterranean basin, and as a traditional medicine, it is applied as a virucidal, fungicidal, and bactericidal agent [119,120]. Its aqueous extract is widely used in this region as an anti-diabetic remedy for its hypoglycemic effect [121]. Remo et al. investigated the aqueous extract of *S. lavandulifolia* Vahl for its α-amylase inhibitory activity as well as its hypoglycemic effect in vivo [96]. The aqueous extract showed significant α-amylase inhibition activity with an IC_50_ of 0.99 mg/mL compared to an IC_50_ of 0.52 mg/mL for acarbose. It also showed a hypoglycemic effect on diabetic rats, with an AUC of 51.94 g/L/h. This study shows that *S. lavandulifolia* is a potential candidate for anti-diabetic drugs (Table 2). 

## 4. Secondary Metabolites Isolated from Various Plant Sources as Potential α-Amylase Inhibitors

**Figure 4:**
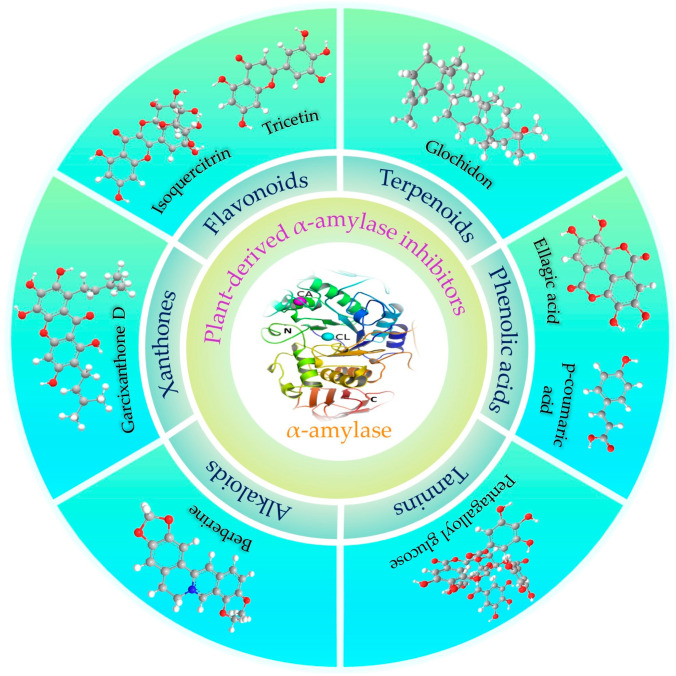
A diagram depicting the numerous plant-derived compounds that are effective α-amylase inhibitors.

Many different plant extracts have been used for therapeutic purposes throughout history, whether on purpose or by accident. There have been reports of numerous plant extracts and their secondary metabolites having anti-diabetic effects, particularly through α-amylase or α-glucosidase inhibition [130,131]. Due to their fewer adverse side effects and easy accessibility, there is a growing interest in developing medicinal plant extracts or the extracted secondary metabolites as an alternative and complementary natural therapeutic for the medical care of diabetes [132,133]. Several secondary metabolite groups, including flavonoids, polysaccharides, phenolic acids, terpenoids, tannins, alkaloids, and xanthones, have been identified as prospective inhibitors of the α-amylase enzyme (Figure 4, Table 3).

### 4.1. Flavonoids

Flavonoids are the most ubiquitous class of secondary metabolites present in plants [164]. They are broadly studied due to their wide range of bioactivities, which include anti-oxidant [77], anti-inflammatory [165], anti-microbial [166], and anti-diabetic properties [167]. Numerous groups of flavonoids have the potential to inhibit α-amylase enzymes due to their non-covalent binding ability to the active site residues of the enzyme [168].

**Figure 5:**
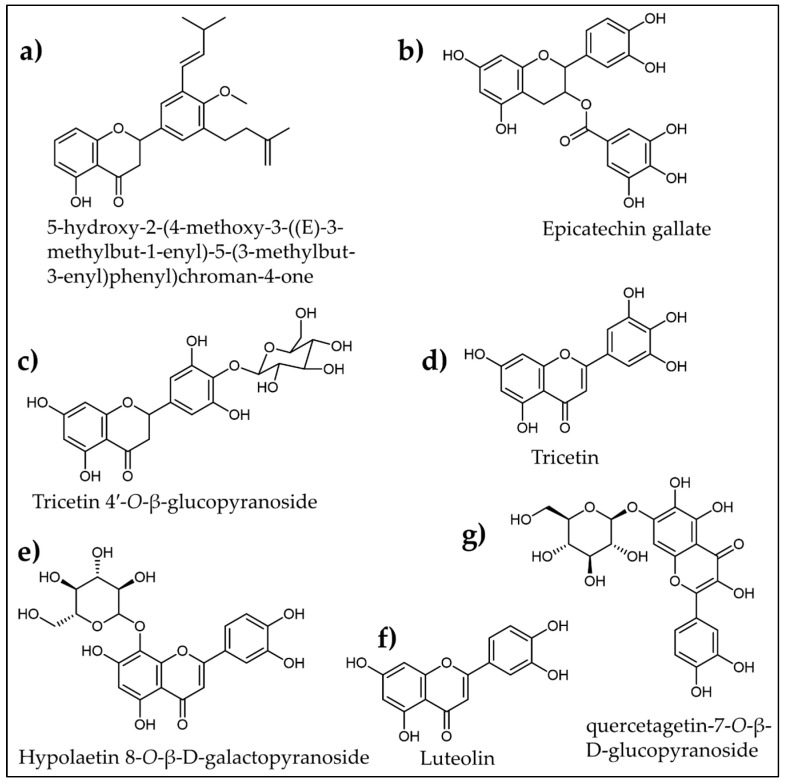
Molecular structures of various plant-derived flavonoids identified as α-amylase inhibitors.

A flavone derivative, 5-hydroxy-2-(4-methoxy-3-((E)-3-methylbut-1-enyl)-5-(3-methylbut-3-enyl)phenyl)chroman-4-one (Figure 5a), identified in *Andrographis echioides* leaf, was found to dramatically inhibit α-amylase (IC_50_ = 3.35 µg/mL) and enhance glucose intake in the 3T3-L1 and L6 cell lines [134]. Another study conducted by Zhang et al. [136] revealed that epicatechin gallate (Figure 5b) (IC_50_ = 0.92 mg/mL) isolated from *Euryale ferox* seed coat possessed good inhibitory effects against α-amylase in comparison to acarbose (IC_50_ = 1.08 mg/mL). Similarly, Wu and Tian [138] isolated a new flavone glycoside, tricetin 4′-*O*-β-glucopyranoside (Figure 5c), along with a known flavone, Tricetin (Figure 5d), from the flowers of *Punica granatum*. Tricetin 4′-*O*-β-glucopyranoside (IC_50_ = 1.17 mg/mL) and Tricetin (IC_50_ = 0.43 mg/mL) both exhibited α-amylase inhibitory activities comparable to acarbose (IC_50_ = 0.03mg/mL). In addition, a new α-glucosidase and α-amylase inhibitor flavonoid named hypolaetin 8-*O*-β-D-galactopyranoside (Figure 5e) was isolated from the leaves of *Thymelaea tartonraira* [140].

Yang et al. [135] separated luteolin (Figure 5f) from *Taraxacum mongolicum,* which inhibits the α-amylase enzyme (IC_50_ = 42.33 µg/mL). From the molecular docking studies, they found that luteolin restricts the bioactivity of α-amylase by making a stable complex with the enzyme through Van der Waals forces, hydrogen bonding, and hydrophobic interaction. In a recent study, Mohamed and his co-workers [139] assessed 12 different flavonoids from *Tagetes minuta* for α-amylase inhibitory activities by in vitro experiments and in silico analyses. Compared to acarbose, quercetagetin-7-*O*-β-D-glucopyranoside (Figure 5g) showed the best in vitro enzyme inhibition (IC_50_ of 7.8 µM), more stability, and the highest binding affinity with the receptor in in silico studies. Such encouraging results are prompting us to further explore the flavonoid molecules and their interaction with α-amylase enzymes in in vivo studies, toxicity assessments, and the development of new anti-diabetic therapeutics.

### 4.2. Terpenoids

Terpenoids are among the diverse classes of natural compounds with potent medicinal qualities such as anti-cancer, anti-inflammatory, and antiviral properties [169]. They are major constituents of essential oils produced by aromatic plants and contribute to their flavor and fragrance [170]. Medicinal plant-derived terpenoids have been found to have promising hypoglycemic effects [171].

**Figure 6:**
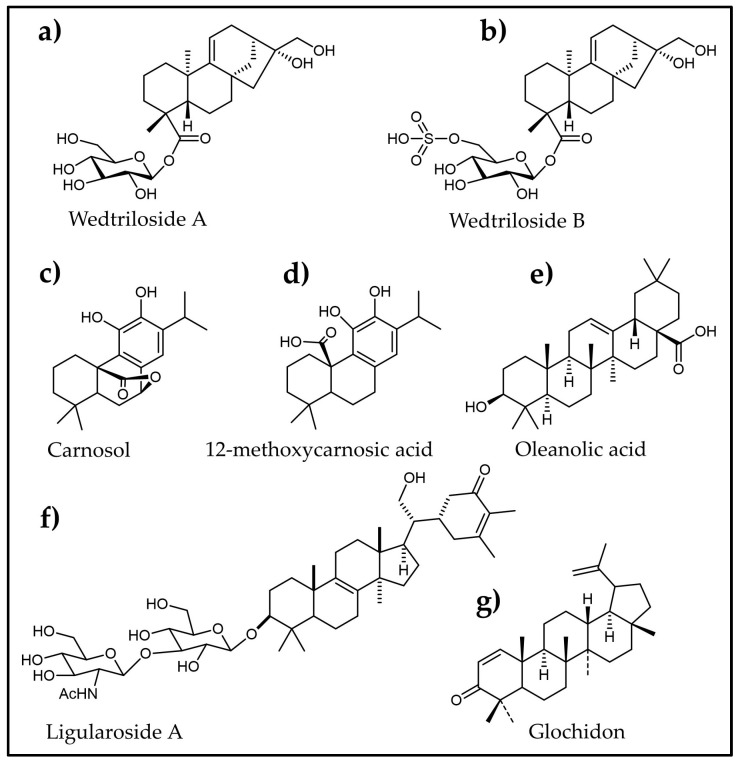
Molecular structures of various plant-derived terpenoids identified as α-amylase inhibitors.

As part of the search for new anti-diabetic compounds from plants, Luyen et al. [141] examined the Vietnamese medicinal plant *Wedelia trilobata*, which was well-known for its efficacy in treating type 2 diabetes. They discovered wedtrilosides A and B (Figure 6a,b), two new ent-kaurane diterpenoids that have α-amylase inhibition activities. Furthermore, abietane diterpenes, carnosol (Figure 6c) and 12-methoxycarnosic acid (Figure 6d), isolated from *Salvia aurita,* revealed strong α-amylase inhibition with an IC_50_ of 19.8 and 16.2 µg/mL, respectively [142]. Similarly, oleanolic acid (Figure 6e) from *Salvia Africana-lutea* exhibited an α-amylase inhibition property with an IC_50_ of 12.5 µg/mL, close to acarbose (IC_50_ = 10.2 µg/mL) [172]. Another new terpenoid saponin molecule, ligularoside A (Figure 6f), was reported for the first time from *Passiflora ligularis* Juss leaves and showed a comparable inhibitory effect over α-amylase with an IC_50_ of 409 μM in an in vitro assay against acarbose, which had an IC_50_ of 234 μM [145].

Verma et al. [144] evaluated the anti-diabetic property of a novel triterpenoid, glochidon (Figure 6g), isolated from *Phyllanthus debilis* by in vitro assays, in vivo trials, and computational studies. In the in vitro assays, glochidon showed excellent α-amylase inhibition (IC_50_ = 38.15 μM), which is almost the same as that of acarbose. Additionally, it showed dose-dependent hypoglycemic effects in STZ-induced diabetic rats. Furthermore, the molecular dynamic simulations demonstrated that glochidon has a higher propensity to interact with the GLUT1 receptor protein. These findings can be utilized in the development of terpenoid-based natural medicine for both the management and avoidance of diabetes and its intricacies.

### 4.3. Polysaccharides

Polysaccharides derived from plants are macromolecules that consist of the same or different monosaccharide units interconnected with α- or β-glycosidic linkages. They have lengthy chains composed of carbohydrate molecules arranged either linearly as in amylose and cellulose or branched as in amylopectin and glycogen [173,174]. The composition, structure, and molecular weight of the polysaccharides vary with the plant species. Polysaccharide composition, molecular weight, linkage types, and chain patterns all influence their physical properties, such as solubility and viscosity, as well as chemical properties affecting pharmacological effects [175,176]. Plant polysaccharides have received a lot of interest in recent years due to their noteworthy bioactivities, non-toxic nature, and suitability for use in medicine [177,178,179]. Recent studies showed that many plant polysaccharides inhibit the α-amylase enzyme and play a crucial role in the treatment of diabetes [180,181,182].

It has been identified that the polysaccharides from *Lycium barbarum* leaves hinder α-amylase enzyme activity in a dose-dependent manner [149]. Another polysaccharide (SGP-1-1), isolated from *Siraitia grosvenorii,* showed α-amylase inhibition with the best inhibition of 61.73% [148]. Similarly, Zeng et al. [128] exploited the red seaweed laver-derived polysaccharide as a hypoglycemic agent. The polysaccharide named PD-1 showed 98.78% inhibition effects on the α-amylase enzyme with an IC_50_ of 12.72 mg/mL. Furthermore, the kinetic studies reveal that PD-1 interacts with the α-amylase enzyme in a competitive manner. Another group of researchers purified two polysaccharides, WSRP-2a (MW 56.8 kD) and WSRP-2b (MW 23.9 kD), from *Rosa Setate x Rosa Rugosa* biomass waste. The inhibition of the α-amylase enzyme by WSRP-2b (IC_50_ = 1.72 mg/mL) was much stronger compared to WSRP-2a (IC_50_ = 3.41 mg/mL), which may be related to changes in molecular weight [147]. Jiang and his coworkers used the ultrasonic-assisted extraction method to extract tamarind (Xyloglucan). When compared to Xyloglucan extracted using the hot water approach, they discovered that the ultrasound-aided extraction method greatly improved its α-amylase inhibitory activity (72.49%) by successfully reducing the viscosity and molecular weight of the compound [150]. In light of these encouraging results, additional research and examinations are needed to determine the correlation between the molecular structure and the anti-diabetic properties of polysaccharides and to create anti-diabetic medications derived from plant polysaccharides.

### 4.4. Phenolic Acids

Phenolic acids are the derivates of cinnamic and benzoic acids and are found in wide varieties of plants, either in free or bound forms [183]. Phenolic acids have been known for diverse bioactivities, including anti-microbial, antioxidant, anti-diabetic, and anti-cancer activities [184]. In carbohydrate metabolism, phenolic acids have been best known for their ability to inhibit the bioactivity of the α-glucosidase and α-amylase enzymes [185].

**Figure 7:**
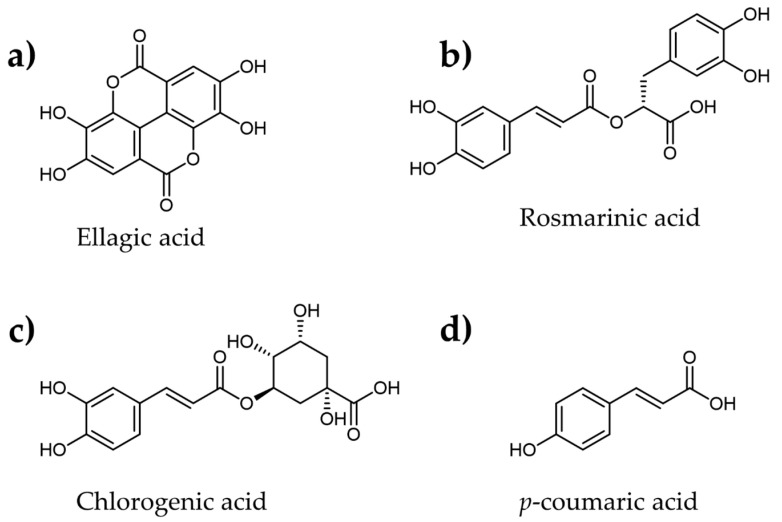
Molecular structures of various plant-derived phenolic acids identified as α-amylase inhibitors.

Wu et al. [74] studied the α-amylase inhibitory effects of ellagic acid (Figure 7a) obtained from corn kernels, which showed better inhibition (IC_50_ = 0.19 mg/mL) than the control (IC_50_ = 0.24 mg/mL). Similarly, Paun et al. [76] isolated rosmarinic acid (Figure 7b) and chlorogenic acids (Figure 7c) from *Anchusa officinalis*. Among these phenolic acids, rosmarinic acid (IC_50_ = 0.92 μg/mL) showed almost 20 fold higher inhibitory effects on the α-amylase enzyme than the standard sample (IC_50_ = 17.68 μg/mL). Likewise, another phenolic acid, p-coumaric acid (Figure 7d), identified in *Agave americana* L., showed considerable inhibition of the human α-amylase enzyme with an IC_50_ of 10.16 μM, which was around 2.3 times higher than the control [137]. In addition, the kinetic studies revealed that p-coumaric acid functions as a competitive α-amylase enzyme inhibitor. To fully realize the potential of phenolic acid compounds as a novel therapeutic for treating diabetes, more study is required to evaluate their efficacy, bioavailability, and safety.

### 4.5. Tannins

Tannins are polyphenolic biomolecules that have the tendency to precipitate proteins in water. They are widely available in many plant species and act as growth regulators and protectors against plant predators [186]. In traditional folk medicine, a variety of tannin-rich plants are frequently used by diabetic patients to treat diabetes mellitus and its associated issues [187].

**Figure 8:**
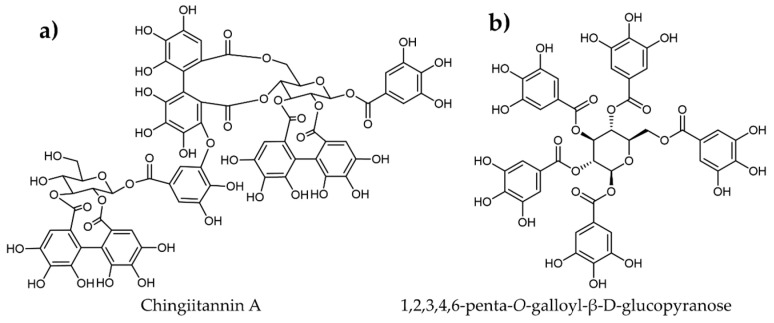
Molecular structures of various plant-derived tannins identified as α-amylase inhibitors.

Wang et al. [153] studied the in vitro α-amylase inhibition of Chinese bayberry leaf-derived proanthocyanidins (BLPs) and found that BLPs could retard the activity of an α-amylase enzyme using a mixed-type inhibition mode with an IC_50_ of 3.07 μg/mL. Chen et al. [151] identified twenty-five major ellagitannins from the unripe fruit of *Rubus chingii* Hu. Among them, chingiitannin A (Figure 8a) demonstrated around seven times higher inhibition with an IC_50_ of 4.52 μM compared to acarbose (IC_50_ = 35.71 μM). Moreover, molecular docking studies revealed that chingiitannin A binds at the allosteric site and primarily bonds with the enzymes through hydrogen bonds. Furthermore, chingiitannin A was non-toxic and improved glucose absorption. Similarly, 1,2,3,4,6-penta-*O*-galloyl-β-D-glucopyranose (Figure 8b), from the leaves of *Rhus coriaria*, was reported to exhibit higher inhibitory effects on an α-amylase enzyme with an IC_50_ of 6.32 μM than acarbose (IC_50_ = 10.69 μM) [70]. However, more scientific analysis is necessary to determine the in vivo anti-diabetic effect of these tannins, and an assessment of toxicity should be performed in order to design therapeutic biomolecules with no or very few side effects.

### 4.6. Miscellaneous Secondary Metabolites as α-Amylase Inhibitors

Plants possess various bioactive compounds with different structural moieties, which demonstrated efficient α-amylase inhibition. For example, Thuy and his co-workers isolated three new pregnane glycosides from *Dregea volubilis* leaves, among which Drevoluoside Q exhibited significant inhibitory effects against α-amylase (IC_50_ = 51.3 µM), which is comparable to acarbose (IC_50_ = 36.3 µM) [157]. Likewise, phytochemical studies of another plant species, *Gymnema sylvestre*, resulted in the separation of five new pregnane glycosides, known as gymsylodide A, gymsylodide B, gymsylodide C, gymsylodide D, and gymsylodide E. Among them, gymsylodides B-E were found to have considerable α-amylase inhibitory action, with half-maximal inhibitory concentrations ranging from 113.0 to 176.2 µM [158].

**Figure 9:**
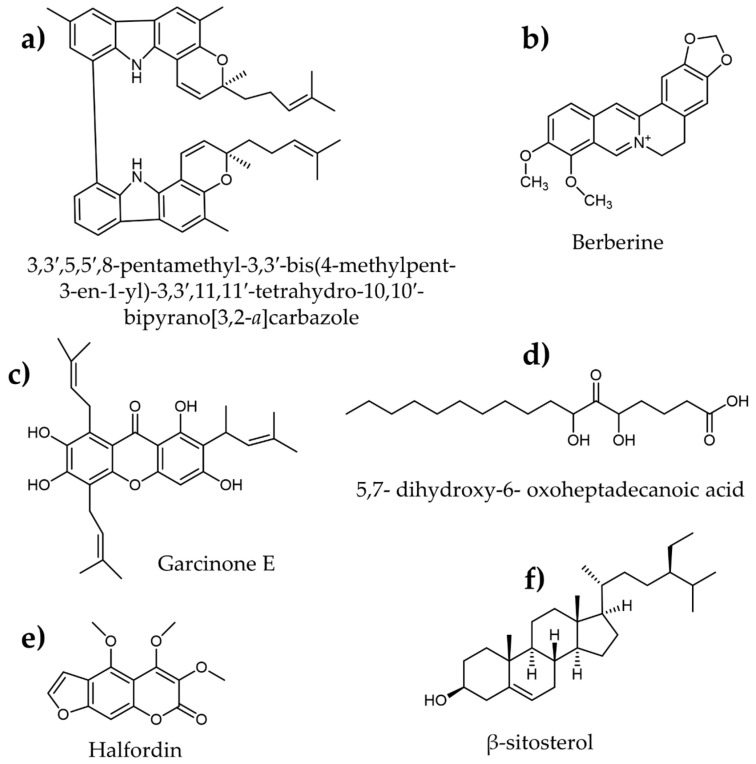
Molecular structures of various plant-derived secondary metabolites identified as α-amylase inhibitors.

An alkaloid, 3,3′,5,5′,8-pentamethyl-3,3′-bis(4-methylpent-3-en-1-yl)-3,3′,11,11′-tetrahydro-10,10′-bipyrano[3,2-*a*]carbazole (Figure 9a), was identified from *Murraya koenigii* leaves, which displayed significant in vitro *α*-amylase inhibition with an IC_50_ of 30.32 ppm [155]. Similarly, another known alkaloid, berberine (Figure 9b), was isolated from the *Cardiospermum halicacabum* plant and found to retard the activity of α-amylase by 72% at 10 μg/mL. In addition, in silico investigations indicated that berberine adheres to the enzyme’s active site [156].

Another class of bioactive molecules, xanthones named garcixanthone D and garcinone E (Figure 9c), were purified from the pericarp of *Garcinia mangostana* and showed 93.8 and 85.6% inhibition against α-amylase, respectively. The molecular docking studies revealed that these two compounds interact with α-amylase differently than the standard acarbose [160]. Similarly, a new prenylated xanthone, mangoxanthone A, with a moderate α-amylase inhibitory effect (IC_50_ = 22.74 μM), was reported from *Garcinia mangostana* pericarp [159].

Makinde et al. [162] discovered a fatty acid derivative, 5,7-dihydroxy-6-oxoheptadecanoic acid (Figure 9d), from the *Tiliacora triandra* plant that inhibited α-amylase enzymes (IC_50_ = 26.27 μM) more effectively than acarbose (IC_50_ = 177.65 μM). Halfordin (Figure 9e), a coumarin molecule purified from the bark extract of *Melicope latifolia*, was investigated for its potential to inhibit α-amylase enzymes [71] and was shown to be effective (IC50 = 197.53 μM). In addition, in silico investigations revealed a large number of molecular interactions with key α-amylase amino acid residues. Moreover, β-Sitosterol (Figure 9f) was identified as an effective α-amylase inhibitor from *Parthenium hysterophorus* leaf extract [163]. According to the molecular dynamics analyses, β-sitosterol has a larger binding energy than acarbose and the highest stability with α-amylase enzymes.

Investigations into natural compounds with significant α-amylase inhibitory effects are critical since many phytocompounds are consumed in the form of food in our daily lives. Based on findings on their potential effectiveness in inhibiting α-amylase enzymes, bioactive natural compounds are thought to play a very critical role in diabetes treatment. As a result, more research and analyses are needed to assess the toxic effect as well as other drug interactions or unfavorable effects that they might create in in vivo studies. Furthermore, additional human clinical trials are required to validate the safety and anti-diabetic benefits of α-amylase inhibitors derived from various plant species.

## 5. Conclusions

Diabetes is a chronic metabolic illness recognized by persistently elevated blood sugar levels resulting from a decrease in insulin synthesis or a rise in insulin resistance. One preliminary-stage diabetes therapy technique is to lessen post-meal hyperglycemia. This can be accomplished by moderating the bioactivity of α-amylases, carbohydrate-digestive enzymes that hinder glucose absorption in the gut. Consequently, inhibitors of these enzymes limit the rate of glucose absorption, lowering postprandial plasma glucose levels. Growing evidence from traditional and herbal approaches to diabetes management indicates that plant extracts and their chemical components may play a substantial role in the therapeutic management of T2D and its implications. Hence, this review study aimed to summarize the latest and most advanced findings in natural product research that operate as α-amylase enzyme inhibitors. Despite the fact that scientists have put in a lot of effort to identify the inhibitory effects of various plant extracts and natural compounds against the α-amylase enzyme, there are still some gaps. The majority of research focuses on in vitro investigations of the inhibitory effect of plant extracts and the identification of bioactive molecules. However, only a very limited amount of additional study was conducted for in vivo tests in animals, and human trials were extremely rare. Therefore, more research and scientific analyses are needed to assimilate the pharmacological activity of natural extracts and compounds, the synergistic effects of active compounds with other molecules, and their safer use. Moreover, clinical trials are crucial for reaching precise conclusions on both the safety and effectiveness of administering plant extracts or active compounds for the management of type 2 diabetes. Prospective studies will offer important information for determining the doses of natural extracts and active compounds that will deliver the desired therapeutic advantages while exhibiting little to no adverse side effects. This study explored over fifty extracts obtained with various solvents and over sixty natural ingredients. The insights gained from this review’s findings should help to achieve the ultimate goal of developing new therapeutic drugs with improved effectiveness and safety for the management of T2D or to avoid PPHG.

## Figures and Tables

**Table 1:** In Vitro α-amylase inhibition properties reported from various plant extracts.

Name of Plants	Used Extract/Fraction/Parts	IC_50_	IC_50_ (Control) (Acarbose)	Ref.
*Argania spinosa*	Roasted seeds oil	2.17 ± 0.24 mg/mL	0.41 ± 0.015 mg/mL	[63]
	Unroasted seeds oil	0.78 ± 0.16 mg/mL		
*Melicope glabra* (Blume) T. G. Hartley	Chloroform extracts (leaves)	303.64 ± 10.10 μg/mL	188.6 ± 14.31 μg/mL	[64]
*Aloe megalacantha* Baker	Leaf latex	74.76 ± 1.98 μg/mL	16.49 ± 1.91 μg/mL	[65]
*Aloe monticola* Reynolds		78.10 ± 1.88 μg/mL	16.49 ± 1.91 μg/mL	
*Hedychium coronarium* Koen	Ethyl acetate fraction (rhizomes)	58.15 ± 1.23 μg/mL	-	[66]
*Cichorium endivia*	Hydrophilic fraction (extracted from leaves with ethanol/water)	9.96 μg/mL	10.00 μg/mL	[67]
Brown rice	Brown rice (BR) extract	48.96 ± 0.34%	54.14 ± 0.35%	[68]
	BR/Vanillin	99.32 ± 1.18%	86.48 ± 0.71%	
	BR/Vanillyl alcohol	96.55 ± 0.12%		
Biyun no.7 (Kidney bean)	Aqueous extract	1.659 ± 0.050 U/g DW	-	[69]
*Rhus coriaria* L.	Ethyl acetate sub-extract (leaves)	20.810 ± 0.747 μg/mL	26.993 ± 0.797 μg/mL	[70]
*Melicope latifolia*	Chloroform extract (bark)	1464.32 μg/mL		[71]
*Arachis hypogaea*	Ethanol extract (peanut seeds)	0.61 μg/mL	0.32 μg/mL	[72]
*Backhousia citriodora*	Essential oil	0.49 mg/μL	-	[73]
*Rosmarinus officinalis*	Essential oil of rosemary plus	0.45 mg/μL		
*Mentha piperita*	Essential oil (leaf)	0.41 mg/μL		
*Origanum vulgare*	Essential oil (leaf-phenol type)	0.41 mg/μL		
*Quercus variabilis* Blume	Free polyphenol extract	5.25 ± 0.57 mg/mL	0.24 mg/mL	[74]
	Bound polyphenol extract	1.37 ± 0.11 mg/mL		
*Centaurea pterocaula* Trautv	Essential oil (aerial part)	79.66 ± 0.43 µg/mL	11.6 ± 0.18 µg/mL	[75]
*Anchusa officinalis*	Crude extract	954.16 ± 7.46 µg/mL	17.68 ± 1.24 µg/mL	[76]
*Melilotus officinalis*	Crude extract	1.32 ± 0.08 µg/mL		
*Clausena indica*	Hexane extract	1.37 ± 0.01 mg/mL	0.07 ± 0.00 mg/mL	[77]
	Ethyl acetate extract	8.56 ± 0.24 mg/mL		
*Prosopis cineraria* (L.)	n-Butanol fraction (pods)	22.01 ± 0.92 μg/mL	39.26 ± 2.19 μg/mL	[78]
	Ethyl acetate fraction	28.23 ± 1.06 μg/mL		
*Senna auriculata* (L.) Roxb.	Methanolic extract (leaves)	49.45 μg/mL	-	[79]
*Terfezia claveryi*	Methanol extract	38.7 μg/mL	45.3 μg/mL	[80]
*Rhododendron arboreum* Sm.	Methanol extract	51.1%	-	[81]
*Phragmites karka* (Retz.)	Dichloromethane fraction (aerial part)	2.05 mg/mL	- -	[82]
	n-Hexane fraction (aerial part)	2.08 mg/mL		
*Morus nigra*	Hexane fraction (leaves)	13.05 mg/mL	0 0.21 mg/mL	[83]
*Salaria basilisca*	Protein hydrolysates (peptide fraction F1)	71 μg/mL	14 μg/mL	[84]
*Leucaena leucocephala* (Lam.) De Wit	Ethanol extract (leaves)	288.01 μg/mL	252.59 μg/mL	[85]
*Bergenia pacumbis*	Methanol extract	14.03 ± 0.04 μg/mL	20.12 ± 0.12 μg/mL	[86]
*Nonea obtusifolia* (Wild.) DC.	Acetone extract	25.7 ± 0.08 μg/mL	28.18 ± 1.22 μg/mL	[87]
*Morus alba Linn*	Leaves	74.76 ± 6.76 μg/mL	35.34 ± 4.87 μg/mL	[88]
*Phylanthus emblica* L.	Methanolic extract (leaves)	98.37 ± 1.09%		[89]
*Catunaregam spinosa*	Dichloromethane fraction (bark)	77.17 ± 1.75 μg/mL	6.34 ± 0.07 μg/mL	[90]
*Chenopodium album* L.	Flavonoid fraction (aerial part)	122.18 ± 1.15 μg/mL	812.83 ± 1.07 μg/mL	[91]
*Solanum virginianum*	Aqueous extract (fruits)	54.12 ± 0.44–86.80 ± 0.27%	58.36 ± 0.30–88.24 ± 0.16%	[92]
	Ethanolic extract (fruits)	23.07 ± 0.47–81.61 ± 0.43%		
*Eucalyptus globulus*	Ethanol extract (Hexane defatted)	23.6 ± 1.2 μg/mL	5.2 ± 1.3 μg/mL	[93]
	Ethanol extract (non-defatted)	14.8 ± 1.2 μg/mL		
*Maesobotrya dusenii* Hutch.	Crude methanol extract	24 μg/mL	28 μg/mL	[94]
*Veronica biloba*	Aqueous extract	110.25 μg/mL	138.79 μg/mL	[95]
	Ethyl acetate extract	121.09 μg/mL		
	Dichloromethane extract	123.68 μg/mL		
*Salvia lavandulifolia* Vahl	Aqueous extract	0.99 ± 0.00 mg/mL	0.52 ± 0.01 mg/mL	[96]
*Moringa oleifera*	Methanolic crude extract (leaves)	65.6 ± 4.93%	-	[97]
*Ziziphus mucronata*	Acetone extract	0.62 mg/mL	0.42 mg/mL	[98]
*Englerophytum magalismontanum*	Methanol fraction (leaves)	10.76 ± 1.33 µg/mL	1.24 ± 1.64 µg/mL	[99]
*Achyranthes aspera*	Crude extract	97.60 ± 1.11 μg/mL	68.13 ± 0.46 μg/mL	[100]
*Catharanthus roseus*		94.05 ± 1.18 μg/mL		
*Pterocarpus marsupium*	Methanolic extract	158.663 ± 10.986 μg/mL	56.060 ± 4.465 μg/mL	[101]
*Rubus corchorifolius* L.	70% ethanolic extract (leaf tea)	1.26 ± 0.03 mg/mL	5.12 ± 0.42 mg/mL	[102]
	70% methanolic extract (leaf tea)	1.47 ± 0.05 mg/mL		
	Aqueous extract (leaf tea)	4.39 ± 0.17 mg/mL		
*Sterculia nobilis* Smith	Ethyl acetate fraction (pericarp)	13.550 ± 0.230 μg/mL	19.45 ± 0.26 μg/mL	[103]

**Table 2:** List of in vivo effects of α-amylase inhibitors reported from several plant extracts.

Plant Extracts	Dosage of Plant Extract Used	Experimental Animals	Types of Diabetes Induction	Administration Route	Diagnostic Criteria	Inference	Ref.
*Oxalis pes-caprae* Methanolic extract	150 mg/kg BW	Swiss albino mice	Alloxan-induced diabetes	Intraperitoneal injection	Fasting blood glucose (FBG) level, body weight	Hypoglycemic	[122]
*Cardamine hirsuta* Linn Hydro-methanolic extract	125, 250, and 500 mg/kg BW	Male Sprague Dawley (SD) rats	High-fat diet (HFD), Streptozotocin (STZ)-induced diabetes	Oral and intraperitoneal injection	FBG level	Hypoglycemic, dose-dependent	[123]
*Trachinotus ovatus* Protein hydrolysates	100, 500, and 1000 mg/kg BW	Male Kunming mice	STZ-induced diabetes	Intraperitoneal injection	FBG level	Hypoglycemic, dose-dependent	[124]
*Sorbaria tomentosa* Lindl. Rehder Methanolic extract	150 and 300 mg/kg BW	Rats	Alloxan-induced diabetes	Intraperitoneal injection	FBG level	Hypoglycemic	[125]
*Chenopodium album* L. Flavonoid fraction	500 mg/kg BW	SD rats	HFD-STZ-induced diabetes	Oral and intraperitoneal injection	Glucose, cholesterol, and triglyceride levels	Hypoglycemic	[91]
*Terfezia claveryi* Methanolic extract	200 mg/kg BW	Male Wistar albino rats	STZ-induced diabetes	Intraperitoneal injection	FBG level	Hypoglycemic time-dependent	[80]
*Salvia lavandulifolia Vahl* Aqueous extract	400 mg/kg BW	Normal rats	D-glucose, 2 g/kg	Oral	Oral glucose tolerance test (OGTT) Blood glucose level	Hypoglycemic	[96]
*Artemisia absinthium* L. Aqueous extract	200 mg/kg BW	Wistar rats	Alloxan-induced diabetes	Oral	Postprandial blood glucose (PBG) level	Hypoglycemic	[126]
*Ammodaucus leucotrichus* Coss. and Durieu Aqueous extract	150 mg/kg BW	Wistar albino rats	Alloxan-induced diabetes	Oral	OGTT	Hypoglycemic	[127]
*Porphyra* spp. Polysaccharides	100 mg/kg BW	Rats	-	Oral	PBG level	Hypoglycemic	[128]
*Prosopis cineraria* Ethyl acetate fraction/n-Butanol fraction	250, 500, 1000 and 2000 mg/kg BW	Swiss albino mice	Sucrose tolerance test (OSTT)	Oral	Serum glucose concentration	Hypoglycemic	[78]
*Allium sativum* L. Polysaccharides	1.25, 2.5, and 5 g/kg BW	Male Kunming mice	STZ-induced diabetes	Intraperitoneal injection	OGTT, FBG	Hypoglycemic	[129]

**Table 3:** List of α-amylase inhibitors (in vitro) isolated from diverse plant species.

Class of Compounds	Bioactive Compound	Source (Plant’s Name)	IC_50_	IC_50_ (Control) (Acarbose)	Ref.
Flavonoids	5-hydroxy-2-(4-methoxy-3-((E)-3-methylbut-1-enyl)-5-(3-methylbut-3-enyl)phenyl)chroman-4-one	*Andrographis echioides*	3.357 µg/mL		[134]
	Luteolin	*Taraxacum mongolicum*	42.33 ± 0.82 μg/mL	-	[135]
	Isoquercitrin	*Melilotus officinalis*	9.65 ± 0.43 μg/mL	17.68 ± 1.24 μg/mL	[76]
	Epicatechin gallate	*Euryale ferox* (seed coat)	0.92 mg/mL	1.08 mg/mL	[136]
	Puerarin	*Agave americana* L.	3.87 μM		[137]
	Tricetin	*Punica granatum*	0.43 ± 0.12 mg/mL	0.038 ± 0.017 mg/mL	[138]
Flavonoid glucoside	Tricetin 4′-*O*-β-glucopyranoside		1.17 ± 0.32 mg/mL		
	Rutin	*Melilotus officinalis*	11.42 ± 0.62 μg/mL	17.68 ± 1.24 μg/mL	[76]
	quercetagetin-7-*O*-β-D-glucopyranoside	*Tagetes minuta* L.	7.8 µM	7.1 µM	[139]
	luteolin-7-*O*-α-L-rhamnoside	*Phylanthus emblica* L.	89.79%	-	[89]
	hypolaetin 8-*O*-β-D-galactopyranoside	*Thymelaea tartonraira* (leaves)	46.49 ± 2.32 μg/mL	0.44 ± 0.022 μg/mL	[140]
Ent-kaurane diterpenoids	Wedtriloside A	*Wedelia trilobata* (leaves)	112.20 ± 2.87 μg/mL	-	[141]
	Wedtriloside B		87.10 ± 1.89 μg/mL		
Abietane diterpene	Carnosol	*Salvia aurita* (aerial part)	19.8 ± 1.4 µg/mL	10.2 ± 0.6 µg/mL	[142]
	12-methoxycarnosic acid		16.2 ± 0.3 µg/mL		
Pentacyclic triterpenoid	Oleanolic acid	*Xylopia aethiopica* (Dunal) A. Rich. (fruit)	89.02 ± 1.12 µM	-	[143]
Triterpenoid	Glochidon	*Phyllanthus debilis*	38.15 ± 1.40 μM	33.68 ± 3.12 μM	[144]
Triterpenoid saponin	Ligularoside A	*Passiflora ligularis* Juss (leaves)	409.8 ± 11.4 μM	234.1 ± 15.9 μM	[145]
Phenylpropanoids	Jionoside D	*Clerodendrum infortunatum* L.	3.4 ± 0.2 μM	5.9 ± 0.1 μM	[146]
Polysaccharides	WSRP-2a	*Rosa setate × Rosa rugosa* (waste biomass)	3.41 mg/mL	0.57 mg/mL	[147]
	WSRP-2b		1.72 mg/mL		
	PD-1	*Porphyra* spp.	12.72 mg/mL		[128]
	SGP-1-1	*Siraitia grosvenorii*	61.73% at 1 mg/mL		[148]
	LLP50 (polysaccharides fraction)	*Lycium barbarum* (leaves)	1.659 mg/mL	0.0002 mg/mL	[149]
	Xyloglucan	*Tamarindus indica* L	72.49 ± 0.84%	92.49 ± 1.97%	[150]
Phenolic acid and its derivatives	Ellagic acid	*Quercus variabilis* Blume	0.19 ± 0.02 μg/mL	0.24 μg/mL	[74]
	Rosmaric acid	*Anchusa officinalis*	0.92 ± 0.07 μg/mL	17.68 ± 1.24 μg/mL	[76]
	Chlorogenic acid		1.84 ± 0.05 μg/mL		
	p-Coumaric acid	*Agave americana* L.	10.16 μM	-	[137]
Ellagitannins	Chingiitannin A	*Rubus chingii* Hu (unripe fruit/n-BuOH fraction)	4.52 ± 0.30 μM	35.71 ± 4.93 μM	[151]
	Lambertianin A		10.32 ± 0.10 μM		
	Sanguiin H-6		11.00 ± 0.21 μM		
Gallotanins	1,2,3,4,6-penta-*O*-galloyl-β-D-glucopyranose	*Rhus coriaria* (leaves)	6.32 ± 0.18 μM	10.69 ± 0.50 μM	[70]
Dihydrostilbene glycosides	Sasastilboside A	*Camellia sasanqua* Thunb (leaves)	53.7 ± 1.6 μM	-	[152]
Proanthocyanidins	Chinese bayberry leaves proanthocyanidins	*Myrica rubra* Sieb. et Zucc. (leaves)	3.075 ± 0.073 μg/mL	-	[153]
Phenolic	Dehydrodieugenol B	*Ocimum tenuiflorum*	29.6 μM	13.85 μM	[154]
Coumarin	Halfordin	*Melicope latifolia*	197.53 µM	282.39 ± 8.14 µM	[71]
Alkaloids	3,3′,5,5′,8-pentamethyl-3,3′-bis(4-methylpent-3-en-1-yl)-3,3′,11,11′-tetrahydro-10,10′-bipyrano[3,2-a]carbazole	*Murraya koenigii* (L.)	30.32 ± 0.34 ppm	-	[155]
	Berberine	*Cardiospermum halicacabum*	72% at 10 μg/mL		[156]
Pregnane glycosides	Drevoluoside Q	*Dregea volubilis* (leaves)	51.3 ± 2.1 µM	36.3 ± 0.5 µM	[157]
	Gymsyloside B	*Gymnema sylvestre* (leaves)	175.8 ± 2.3 µM	72.4 ± 0.8 µM	[158]
	Gymsyloside C		162.2 ± 2.7 µM		
	Gymsyloside D		113.0 ± 0.7 µM		
Prenylated xanthones	Mangoxanthone A	*Garcinia mangostana* (pericarp)	22.74 ± 2.07 µM	-	[159]
Xanthone	Garcixanthone D	*Garcinia mangostana* (pericarp)	93.8%	96.4%	[160]
	Garcinone E		85.6%		
Xanthophyll	Fucoxanthin	*Phaeodactylum tricornutum*	28.38 ± 0.67 mmol/L	25.01 ± 1.38 mmol/L	[161]
Fatty acid	5,7-dihydroxy-6-oxoheptadecanoic acid	*Tiliacora triandra*	26.27 ± 1.11 μM.	177.65 ± 0.88 μM	[162]
Phytosterols	β-Sitosterol	*Parthenium hysterophorus* (leaves)	42.30% (at 400 µg/mL)	-	[163]
